# Brandy and Throat Disease

**Published:** 1854-04

**Authors:** 


### BRANDY AND THROAT DISEASE.

In several instances persons have applied to me who had been advised to take brandy freely for a throat affection. None but an ignorant man or a drunkard would give such advice; it is warranted by no one principle in medicine, reason, or common sense. The throat is inflamed, the brandy inflames the whole body, and the throat affection, being less urgent from its being scattered over a smaller surface, is less felt, and the excitement of the liquor gives a general feeling of wellness, until the system becomes accustomed to the stimulus, and then the throat, body and the man, all the more speedily go to ruin together.

I have in my mind, while writing these lines, the melancholy history of two young men, one from Kentucky and the other from Missouri, who were advised to drink brandy freely, three times a day, for a throat complaint; one of them, within a year, became a confirmed drunkard and lost his property, and will leave an interesting family in want within another year. The other was one of the most high-minded, honorable young men I have lately known; he was the only son of a widow, and she was rich; *within six months* he became a regular toper, lost his business, spent all his money, and left secretly for California, many thousands of dollars in debt.

